# Experimental assessment of interactions between marine bacteria and model protists: from predator-prey relationships to bacterial-mediated lysis

**DOI:** 10.1128/aem.00929-25

**Published:** 2025-05-30

**Authors:** Diana Axelsson-Olsson, Nikolaj Gubonin, Stina Israelsson, Jarone Pinhassi

**Affiliations:** 1 Centre for Ecology and Evolution in Microbial model Systems - EEMiS, Linnaeus University 4180, Kalmar, Sweden; University of Delaware, Lewes, Delaware, USA

**Keywords:** marine bacteria, unicellular eukaryotes, protists, interactions, lysis, predatory, commensal, amoeba

## Abstract

Bacteria in aquatic environments are a principal food source for predatory protists. Whereas interactions between bacteria and protists are recognized to determine the pathogenesis and epidemiology of several human pathogens, few studies have systematically characterized the interactions between specific aquatic bacteria and protists beyond the prey-predator relation. We, therefore, surveyed individual co-cultures between 18 different genome-sequenced marine bacteria with known virulence gene repertoires and three model protist species widely used for assessing bacteria-protist interactions. Strikingly, 10, 5, and 3 bacterial isolates were capable of lysing the protists *Acanthamoeba polyphaga, Tetrahymena pyriformis,* and *Euglena gracilis*, respectively. A majority of the bacteria were able to grow and/or maintain viable populations in the presence of viable protists. Some bacteria survived longer with viable protists but not heat-killed protists and were observed in protist vacuoles. In this respect, marine bacteria are similar to several protist-dependent human pathogens, including *Legionella*. Analyses of growth patterns in low-nutrient media showed that co-cultivation with *A. polyphaga* allowed one bacterial strain to overcome nutritional stress and obtain active growth. Five isolates depended on viable amoebae to grow, notwithstanding nutrient media status. The remarkable capability of these marine bacteria to survive encounters with, and even actively kill, model predatory protists under laboratory conditions suggests that diverse bacterial defense strategies and virulence mechanisms to access nutrients may be important in shaping microbial interactions. If verified with native marine and freshwater populations, the diversity of interactions uncovered here has implications for understanding ecological and evolutionary consequences of population dynamics in bacteria and protists.

**IMPORTANCE:**

The microbiome constitutes the base of food webs in aquatic environments. Its composition partly reflects biotic interactions, where bacteria primarily are considered prey of predatory protists. However, studies that focus on one or a few species have shown that some bacteria have abilities to escape grazing and may even be capable of lysing their protist predators. In this study, we substantially extend these findings by systematically investigating interactions among multiple taxa of both bacteria and protists. Our results show that marine bacteria display a wider and more complex range of interactions with their predators than generally recognized—from growth dependency to protist lysis. Given that such interactions play key roles in the pathogenesis and epidemiology of several human pathogens, our findings imply that bacterial virulence traits can contribute to defining the structure and ecology of aquatic microbiomes.

## INTRODUCTION

Bacteria constitute the basis for microbial food webs in the aquatic environment, being the principal food source for predatory unicellular eukaryotic organisms—protists—like heterotrophic flagellates. In most environments, however, in addition to the bacteria being grazed by bacterivores, bacteria and protists interact in multiple ways as a consequence of a very long shared evolutionary history. Interkingdom interactions between these microbes include parasitism, mutualism, and symbiosis (1). From the bacterial perspective, this can result in prolonged survival, enhanced dissemination, gene transfer, and even altered pathogenicity (2–5). Some bacteria are resistant to protist grazing (6, 7) and may in fact produce toxic compounds that are lethal for heterotrophic and phototrophic protists (8–12). Hence, interactions between these microorganisms could be an important factor in the ecology of natural waters, not only from a grazing perspective, but also from a perspective where bacteria could control protist viability and abundance. For example, it has been proposed that algicidal bacteria can be involved in terminating algal blooms (13). The mechanisms behind such potential effects are largely unknown and knowledge on the prevalence and function of interactions between bacteria and protists in marine environments, other than the latter preying on the former, remains limited.

From a human health perspective, in contrast, the mechanisms behind different types of interactions between bacteria and protists are well studied since they in several cases are crucial for bacterial pathology and epidemiology; reviewed by Greub and Raoult (14) and Balczun et al. (15). As a prime example, co-culture experiments of the human pathogen *Legionella pneumophila* with the amoeba *Acanthamoeba castellanii* show that bacterial survival and dissemination critically depend on the association of bacteria with the amoeba. Moreover, the interaction also enhances the ability of the bacteria to invade macrophages (3) and augments intracellular survival in monocytes (16). Also, for the pathogen *Mycobacterium avium,* the virulence against human macrophages is significantly enhanced by replication in *A. castellanii* (17). In a similar manner, interactions with the amoeba *A. polyphaga* elevate cytotoxicity and pro-inflammatory potential of bacteria in murine macrophages (18). However, the opposite situation is observed for *Francisella novicida* where the interaction with *A. castellanii* results in a decreased infectivity of mice (19). These studies show that, in addition to bacteria being grazed by bacterivores, microbial interactions between protists and bacteria may determine the pathogenicity and epidemiology of bacteria.

Similar to the medically important amoebae examples above, protists in aquatic environments may be potential promoters of bacterial survival, persistence and growth, possibly offering shelter, site for reproduction, and increased dissemination (2, 20–23). Currently, novel culturing strategies and high-throughput sequencing analyses are casting light on the extensive phylogenetic diversity and phenotypic characteristics of protists dominant in the sea and in freshwater. Pathogenic bacteria, capable of benefiting from interactions with protists (24, 25), have been found in fish and aquatic environments (26). Such strains may contribute to serious economic losses in marine and freshwater ecosystems, impacting both the fish industry and marine recreation activity, as well as possibly being a threat to human health.

Earlier studies have indicated that microbial interactions between bacteria and protists in aquatic environments can be complex and that specific interactions depend on the species pairs under study (8). To exemplify, marine diatom interactions with bacteria are directly linked to the species composition of both algal and bacterial assemblages and to the physiological state of the algae (27, 28). The presence of algicidal bacteria is recognized and has been linked to phytoplankton bloom dynamics (29). However, the prevalence, long-term effects, and molecular mechanisms of interactions between free-living bacteria and protists are poorly understood. One contributing factor to a multitude of interaction modes could be bacterial expression of virulence genes. As shown by Persson et al. (30), virulence gene homologs are abundant in marine bacteria. The functions of such genes in marine bacteria are generally unknown, but their presence indicates that some bacteria may use them to infect or consume eukaryotic cells such as protists. Indeed, this has been shown to be the case with *Pseudomonas aeruginosa*, which uses type III secretion system to kill biofilm-associated amoebae (31). The virulence of this system, with a particular focus on aquatic pathogens, is reviewed by Rahmatelahi et al. (32). In the ocean surface, heterotrophic bacteria are the principal degraders of dissolved organic matter (33). Marine heterotrophic bacteria can be sorted along a continuum from copiotrophs to oligotrophs (34). The larger genomes of copiotrophs allow for a wider array of traits, making them more versatile in adapting to environmental changes. These traits include faster growth, cell motility, signal transduction mechanisms, and defense mechanisms (34). It can, therefore, be expected that bacteria that tend toward copiotrophic life strategies and that encode virulence systems may actively derive nutrients from protists (29, 30). To better understand the implications of interactions between bacteria and protists in aquatic environments, knowledge on the prevalence and nature of these interkingdom relationships is necessary.

The aim of the present study was to survey the interactions between bacteria and protists during long-term co-culturing. Often studies investigate interactions of one or a few model bacteria or protists using laboratory-specific experimental setups, which makes comparisons between studies or taxa difficult. To evaluate the variety of possible interactions among multiple bacteria and protists in a coherent experimental context, we here systematically investigated 18 different marine bacterial species and 3 widely studied model protist species. The protists are found in various aquatic environments although mostly in freshwater. Our previous finding of defined virulence gene repertoires in the genomes of the 18 bacteria (30) was an important motivation to attempt to determine if the bacteria could cause harm to the protists to an extent that is not yet recognized in microbial ecology. The protists studied included *A. polyphaga*, a phagocytic amoeba, well known to harbor bacteria which benefit from this interaction (14, 21, 35). *Acanthamoeba* species have a ubiquitous distribution (36), and *A. polyphaga*, although primarily a freshwater taxon, is also found in marine waters (37). *Euglena gracilis* is a mixotrophic flagellate found in freshwater (38). *Tetrahymena pyriformis,* a ciliate with high grazing capability (39), is similarly a freshwater protist but has also been isolated in saline environments, such as salt marshes (40). All three protists were previously examined in an interaction study with the foodborne pathogen *Campylobacter jejuni* (41). Due to the prominent position of amoeba as widely used model protists for interactions with bacteria, interactions with *A. polyphaga* were investigated in furthest detail. Our null hypothesis was that co-culturing of bacterivorous protists with bacteria would result in equal inhibition of growth of the studied bacteria due to grazing. On the contrary, our results showed that several bacterial species were potent killers of protists, and in several cases, viable protists increased bacterial survival capability.

## RESULTS

### Selective and density-dependent bacterial-mediated lysis of protists

Several bacterial species were observed to have negative effects on the survival of the protists. The ciliate *T. pyriformis* was lysed as an effect of co-culturing with five different bacteria at an initial inoculation ratio of bacteria:protists of 10:1 (Table 1). The flagellate *E. gracilis* was lysed by three different bacterial species (at both high and low inoculation ratio). Interestingly, co-culturing with the bacterial strains *Thalassobaculum* sp. BAL199 and *Vibrio campbellii* AND4 caused decreased and increased green coloration on *E. gracilis*, respectively. Seven bacterial strains caused *E. gracilis* to alter its morphology to a more rounded shape. Ten different bacteria lysed the amoeba *A. polyphaga* when added in the high bacteria:protist ratio (Table 1). Four of these bacteria were also able to lyse the amoeba cells when the bacteria were added in a much lower ratio (1:1,000). Additionally, four bacteria altered the amoebae morphology when added in the high bacteria:protist ratio (10:1), from typically stretched out trophozites to rounded cells typical for stressed amoeba.

**TABLE 1:** Marine bacterial species included in the study and their interactions in co-cultures with *Acanthamoeba polyphaga*, *Tetrahymena pyriformis,* and *Euglena gracilis^d^*

Bacterial strain	*Acanthamoeba polyphaga*					*Tetrahymena pyriformis*				*Euglena gracilis*				Virulence genes^*c*^
	Effect on protista (days)		Bacterial survival (days)		Bacterial presence inside *A. polyphaga*	Effect on protista (days)		Bacterial survival (days)		Effect on protista (days)		Bacterial survival (days)		
	1:1,000^*a*^	10:1^*a*^	Control	Co-culture^*b*^		1:1,000	10:1	Control	Co-culture	1:1,000	10:1	Control	Co-culture	
*Bacteroidetes*														
*Dokdonia donghaensis* MED134	None	Lysis (4)	6	15	Vacuoles	None	None	6	15	None	None	6	3	None
*Polaribacter dokdonensis* MED152	None	None	3	3	Not detected	None	None	6	15	None	Round (5)	6	9	None
*Leeuwenhoekiella blandensis* MED217	None	Round (4)	12	12	Vacuoles, cytoplasm	None	None	6	6	None	None	3	3	AFI
*Eubacterium* sp. SCB49	None	Round (4)	15	15	Cytoplasm	None	None	6	15	None	None	6	15	None
*Flavobacterium* sp. BAL38	Lysis (6)	Lysis (4)	15	15	Cytoplasm	None	None	15	15	Lysis (4)	Lysis (1)	15	15	None
*Pedobacter* sp. BAL39	Lysis (2)	Lysis (6)	15	15	Vacuoles	None	None	15	15	None	Round (4)	15	15	None
***Alphaproteobacteria***														
*Roseobacter* sp. MED193	None	Round (4)	6	15	Cytoplasm	None	None	6	15	None	None	3	3	T4SS, T6SS
*Thalassobaculum* sp. BAL199	None	Lysis (5)	15	15	Not detected	None	None	15	15	Coloration	Coloration	15	15	T6SS
*Loktanella vestfoldensis* SKA53	None	None	12	12	Not detected	None	None	3	15	None	Round (4)	6	15	None
*Sphingomonas* sp. SKA58	Lysis (5)	Lysis (3)	15	15	Vacuoles	None	Lysis (4)	15	15	Lysis (5)	Lysis (1)	15	15	T3SS, T4SS
*Gammaproteobacteria*														
*Neptuniibacter caesariensis* MED92	None	None	6	3	Cytoplasm	None	None	3	3	None	None	3	3	None
*Marinomonas blandensis* MED121	None	None	6	6	Vacuoles	None	None	6	6	None	None	3	3	T6SS
*Vibrio* sp. MED222	Round (5)	Round (4)	6	15	Vacuoles	None	None	6	15	None	Round (5)	3	15	T6SS
*Reinekea blandensis* MED297	None	Lysis (3)	15	15	Vacuoles	None	Lysis (4)	15	15	None	Round (5)	15	15	T6SS
*Vibrio campbellii* AND4	None	Lysis (3)	9	15	Vacuoles, cytoplasm	None	Lysis (5)	15	15	Coloration	Coloration	15	15	T3SS, T6SS, AFI
*Bermanella marisrubri* RED65	None	Lysis (3)	15	15	Vacuoles, cytoplasm	None	Lysis (4)	15	15	None	Round (6)	15	15	T5SS, T6SS
*Stenotrophomonas* sp. SKA14	Lysis (2)	Lysis (1)	15	15	Vacuoles	Lysis (4)	Lysis (1)	15	15	Lysis (4)	Lysis (2)	15	15	T5SS, T6SS
*Photobacterium* sp. SKA34	None	Lysis (5)	6	15	Vacuoles	None	None	15	15	None	Round (4)	15	15	T6SS, PAI

^*a*^ Ratio of inoculum bacteria:protista.

^*b*^ 10:1 bacteria:protista inoculum in all co-cultures.

^*c*^ Data from Persson et al. (2009), AFI, anti-feeding island; PAI, pathogenicity island.

^*d*^ Survival in co-culture, bacteria-induced lysis and other effects, intra-amoebal presence of bacteria in *A. polyphaga,* and the virulence gene repertoire for the bacterial strains are shown.

**Fig 1:**
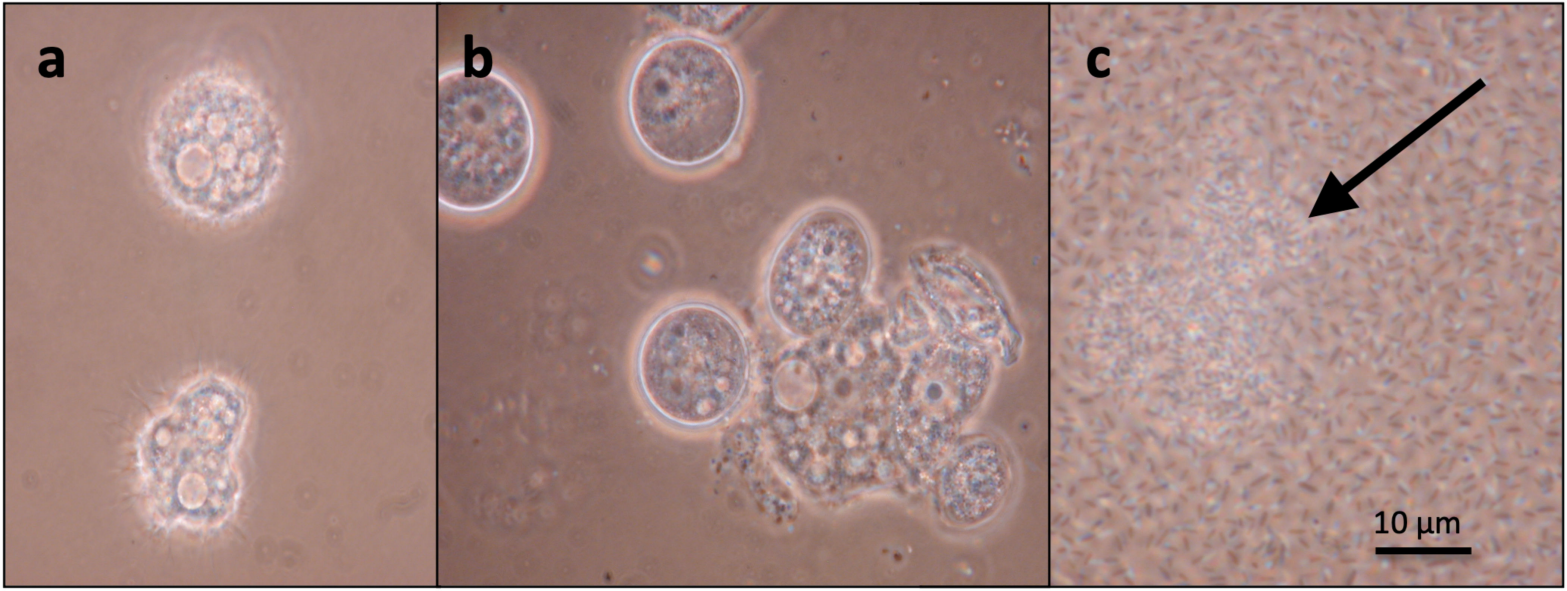
Microscope images of *Stenotrophomonas* sp. SKA14 and amoebae in co-culture. (**a**) 1 h incubation, low bacterial concentration, amoebae in trophozoite form. (**b**) 24 h incubation, moderate bacterial concentration, amoebae start forming rounded cysts. (**c**) One week incubation, rich bacterial concentration, ruptured amoebas (arrow).

There were notable differences between bacteria in the ability to lyse the model protists. The bacterium *Stenotrophomonas* sp. SKA14 (Gammaproteobacteria) was the only species that lysed all three protists (within 1–4 days) regardless of the inoculum ratio (Table 1). As seen in Fig. 1, SKA14 caused *A. polyphaga* to start forming cysts and lyse within 24 h and eventually causing complete lysis within 1 week, while growing to high bacterial concentrations. Also, *Sphingomonas* sp. SKA58 (Alphaproteobacteria) was capable of lysing all three protists although less potently. Co-culturing of *A. polyphaga* and *T. pyriformis* with three Gammaproteobacteria (i.e., MED297, AND4, and RED65) at high inoculum ratio (10:1) caused these protists to lyse, while these bacteria only caused changes in cell morphology of *E. gracilis* (Table 1). *Flavobacterium* sp. BAL38, on the other hand, caused *A. polyphaga* and *E. gracilis* to lyse during co-culture starting with both a high and a low inoculum, while *T. pyriformis* did not indicate any co-culturing effect with this bacterial species.

### Enhanced or maintained bacterial survival in co-culture with protists

Bacterial survival was in several cases stimulated by co-culturing (Table 1). Also, many bacteria did not show any differences in survival time when comparing protist co-culturing with bacterial monoculturing. Most of the bacteria were successfully monocultured in each of the protist-adapted media (Table 1, and Fig. 3 for growth curves in PYG), showing that the marine bacteria were able to grow despite near freshwater salinity levels and variations in carbon quality and quantity. Only in two cases did the protists (*A. polyphaga* and *E. gracilis*) degrade all the bacteria in co-culture (*Neptuniibacter caesariensis* MED92, *Dokdonia donghaensis* MED134). MED134 has previously been observed to be grazed upon by MAST flagellates using fluorescence labeling (42). Most of the bacteria survived through the entire 15-day experiments. Co-culturing with T. pyriformis resulted in a prolonged survival for six bacterial strains (Table 1) as compared to bacterial survival in medium alone. *E. gracilis* caused a prolonged bacterial survival in four bacterial strains (Table 1).

Bacterial co-culturing with *A. polyphaga* prolonged the survival of five bacterial species (Table 1). *Vibrio* sp. MED222 was the only bacterium that showed a prolonged survival when co-cultured with all three protist species, as compared to culturing in protist medium alone. During this experiment, bacterial presence within *A. polyphaga* cells was also assessed. The results showed that 15 out of 18 bacterial species (all except *Polaribacter dokdonensis* MED152, *Thalassobaculum* sp. BAL199, and *Loktanella vestfoldensis* SKA53) were detected inside amoeba cells, either within vacuoles or in the cytoplasm, or in some cases both (Table 1).

**Fig 2:**
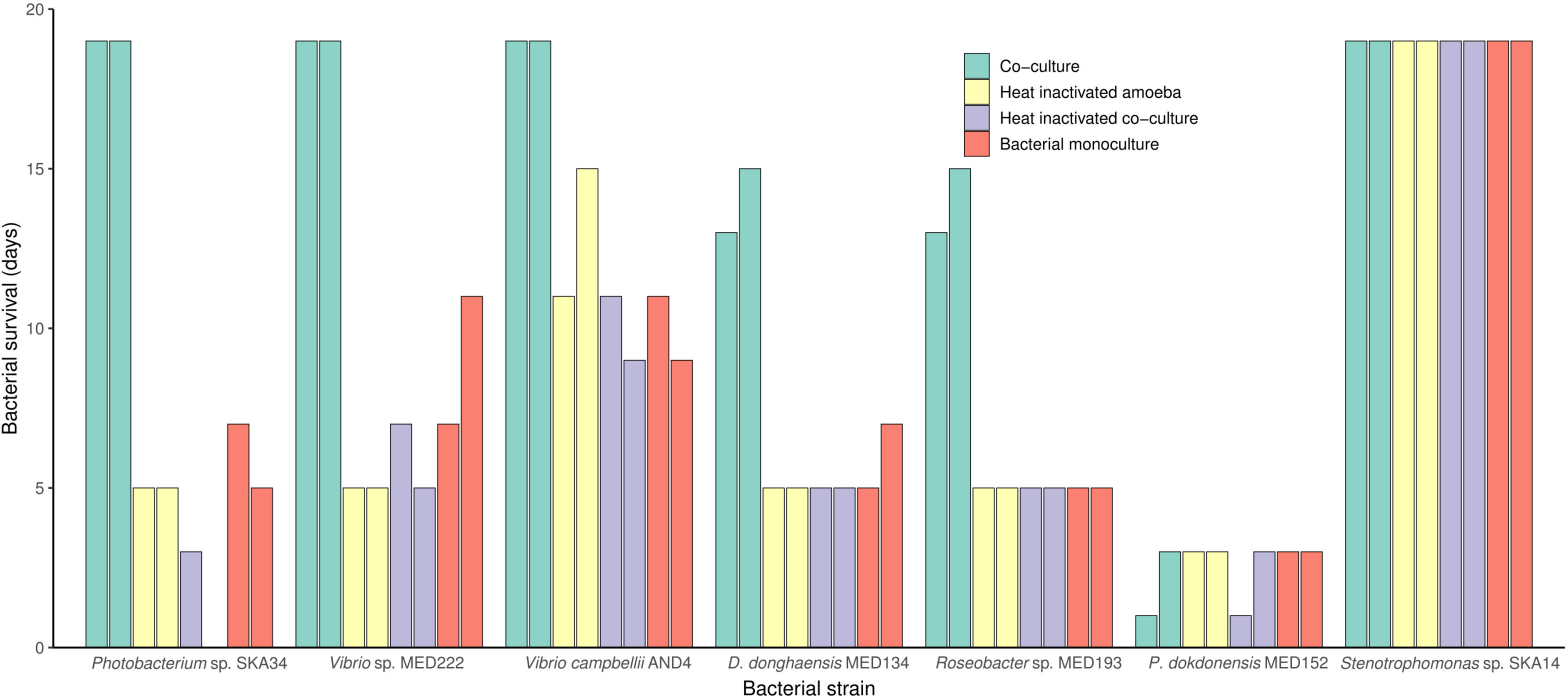
Bacterial survival in co-culture depending on amoeba viability. Bacterial strains were cultured in the presence of viable *A. polyphaga* (green bars), heat-inactivated *A. polyphaga* (yellow bars) or a heat-inactivated previous co-culture of *A. polyphaga,* and the same bacterial strain (purple bars) and compared to bacteria growing in monoculture (red bars). The five strains that showed a dependence on amoeba viability (SKA34, MED222, AND4, MED134, and MED193) are shown as well as two examples of independence of amoeba viability in co-culture (MED152 and SKA14). Survival time of the remaining strains showing amoeba independence is shown in Fig. S1. The bacteria:amoeba ratio was 10:1, and duplicate samples were incubated for a maximum of 19 days. Bacterial viability was tested by plating, initially after 24 h followed by every second day, and shown as mean values. See Table 1 for full species names.

Having found that bacteria and protist co-culturing may result in very different effects (Table 1), interactions between bacteria and *A. polyphaga* were investigated in further detail (Fig. 2, Fig. S1). The importance of amoebae viability was investigated by comparing co-cultures using both viable and heat-inactivated amoebae. Additionally, possible interactions from heat-inactivated previously viable co-cultures were also investigated. Figure 2 shows the different outcomes, represented by the dynamics of seven of the bacterial species, observed with all 18 bacterial strains. Strikingly, five bacterial strains (Fig. 2) all benefited the most from the presence of viable but not dead amoebae, in comparison to bacterial monocultures. The *Photobacterium* sp. SKA34, for example, had a maximum survival time of 19 days in the presence of viable amoeba cells, compared to only 1–6 days when cultured without viable amoebae. Other bacteria did not survive any treatment, as represented by *Polaribacter dokdonensis* MED152, or tolerated all conditions well as represented by *Stenotrophomonas* sp. SKA14 (Fig. 2). Survival times of the remaining 11 strains are shown in Fig. S1.

### Bacterial co-culture with *A. polyphaga* under different nutrient conditions reveals diverse dynamics

**Fig 3:**
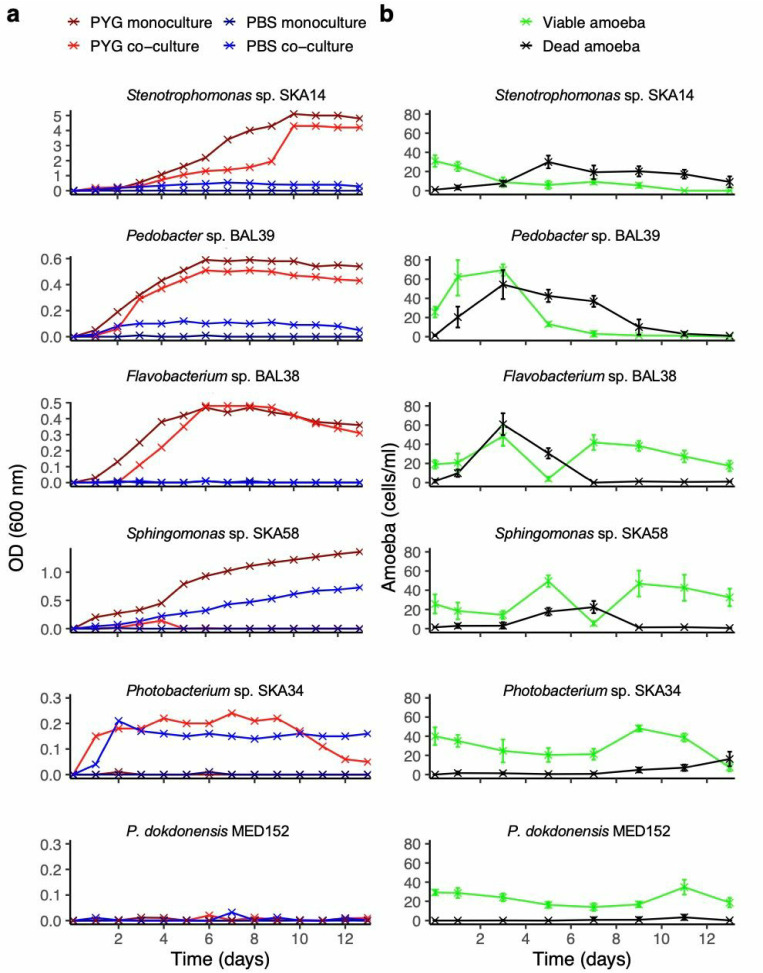
Growth curves of six bacterial strains and *A. polyphaga* viability in co-cultures with respective bacteria. (**a**) The bacteria were cultivated in nutrient-rich media (peptone yeast glucose [PYG]), low-nutrient media (phosphate-buffered saline [PBS]), and in mono- and co-culture with *A. polyphaga*, showing one experimental replicate. (**b**) *A. polyphaga* viability in co-cultures with each of the bacterial strains. Note the different *y*-axis scales of the bacteria growth curves. The bacteria:amoeba ratio was 10:1, and all bacteria cultures started from late exponential growth phase. Bacterial growth and amoeba viability were determined by plating and trypan blue exclusion, respectively. For amoeba viability, error bars display the range across two replicates. Bacterial growth curves of the remaining 12 strains included in the study are shown in Fig. S2.

The effect of nutrient status of culture media on the amoeba-bacteria interactions was investigated by co-culturing in nutrient-rich medium (PYG) and low-nutrient medium (PBS) using bacterial inocula from late exponential growth phase. Monoculturing in PBS did not support either bacterial or amoebal growth (although *A. polyphaga* is known to survive for weeks in PBS), as shown for representative bacterial strains in Fig. 3.

*Stenotrophomonas* sp. SKA14 reached high optical density values (approximately 4–5) when grown in PYG medium for 13 days (Fig. 3a). When grown in PYG co-culture with amoebae, growth was slower for the first 8 days, but from day 10, the OD reached around 4. As bacterial growth progressed, the viability of *A. polyphaga* decreased, and when the bacteria reached stationary phase on day 10, all amoebae had died (Fig. 3b). SKA14 did not grow in PBS alone but showed a minor increase in OD when co-cultured with amoebae in PBS (Fig. 3a).

*Pedobacter* sp. BAL39 interacted with the amoeba in a similar way as SKA14, but differed in that it reached lower maximum OD values, around 0.6, after 6 days, and in that growth was only slightly inhibited by co-culturing with *A. polyphaga* (Fig. 3a). Although BAL39 reached lower OD values, its growth led to death and subsequent disintegration of nearly all *A. polyphaga* cells by day 8 (Fig. 3b). In PBS, co-culturing with amoebae generated a modest growth of BAL39, while no growth was observed in PBS without amoebae (Fig. 3a).

*Flavobacterium* sp. BAL38 growth in co-culture with amoeba in PYG was delayed by approximately 1 day as compared to bacterial monoculture in PYG although both cultures reached OD maxima at nearly 0.5 (Fig. 3a). Although amoeba fitness was clearly negatively affected by co-culturing with BAL38 initially, as shown by a simultaneous increase and then decrease in abundance of both viable and dead amoebae, the amoebae recovered as compared to the amoebae monoculture control (Fig. 3b and a, respectively). The BAL38 bacteria did not grow in PBS, either with or without amoeba.

*Sphingomonas* sp. SKA58 reached an OD value of approximately 1.4 when grown for 13 days in PYG (Fig. 3a). When grown in co-culture with amoeba in PYG media, slow growth to only OD = 0.2 until day 4 was observed, after which the amoeba grazing cleared the cultures of bacteria. The amoeba fitness during the co-incubation in PYG was first positively affected, followed by a drastic viability decline, where the abundance of dead amoebae increased simultaneously, from which the amoebae subsequently managed to recover (Fig. 3b). While SKA58 did not grow in PBS alone, the bacteria grew well in PBS with amoeba, reaching an OD of about 0.7 by the end of the experiment.

*Photobacterium* sp. SKA34 was not capable of growth in either PYG or PBS (Fig. 3a). However, in co-culture with *A. polyphaga* in both PYG and PBS, SKA34 rapidly grew to OD values around 0.2. Thereafter, ODs remained stable in PBS but decreased toward the end of the experiment in PYG. The viability of the amoebae was not substantially affected by co-culturing with SKA34 (Fig. 3b), as compared to the amoebae monoculture control (MED 152, Fig. 3b). Similar dynamics in OD values in PBS and PYG were also observed with five additional bacterial strains (Fig. S2) although two of these strains (*Neptuniibacter caesariensis* MED92 and *Roseobacter* sp. MED193) grew more poorly in co-culture in PBS compared to PYG.

As exemplified by *P. dokdonensis* MED152 (Fig. 3), six bacterial species did not grow either in PYG or in PBS, irrespective of amoeba co-culturing (Fig. S2). Similar dynamics were also observed for seven additional strains (see Fig. S2) although in the case of *D. donghaensis* MED134 some growth but poor survival was observed in amoeba co-culture in PYG. In summary, nine bacterial strains were capable of growth in both PYG and PBS when cultured together with viable (and presumably grazing) amoeba cells. Two of these bacterial strains (*Stenotrophomonas* sp. SKA14 and *Pedobacter* sp. BAL39) were also capable of killing the amoeba cultures completely.

## DISCUSSION

The current work was inspired by studies in medical microbiology where analyses of interactions between bacteria and model protists have provided insights into how microbial interactions influence bacterial pathogenesis and epidemiology (15, 41, 43–46). The potential ecological importance of interactions between bacteria and protists in a marine context, other than bacteria being a primary source of food for protists, is being increasingly acknowledged (8, 29, 47). We surveyed the outcome of interactions between a wide variety of phylogenetically distinct marine bacteria and three different protists under defined experimental conditions, allowing direct comparisons of their performance. This showed what types of interactions occur, how widespread and specialized they may be, and if any of the marine bacteria could gain fitness benefits from interacting with protists. All together, we observed four modes of interactions (beyond bacteria being prey for protists): (i) bacteria lysed the protists, (ii) simultaneous maintenance of bacterial and protist viability, (iii) bacteria utilizing protist-derived nutrients under low-nutrient conditions, and (iv) viable protists required for bacterial growth.

Within an ecological context, an experimental limitation that could affect interpretations of the interaction patterns in this study is the potential disconnect between the natural habitats of the species involved. Specifically, all the bacterial isolates are of marine or brackish origin, while the three protists are primarily freshwater taxa although both *A. polyphaga* and *T. pyriformis* have been found in marine environments with elevated salinity (36, 40). We chose the bacterial model organisms because we had previously sequenced their genomes and had identified virulence genes in several of them (30), and the protists were used because we wanted to draw on the extensive volume of literature that exists for these model protists, especially on *Acanthamoeba*. Also, because we had access to and previous experience of working with the protists used in this study (41, 46, 48), it facilitated the experimental design and interpretation of the results to use them. In fact, there are few model protists of marine origin, with the exception of *Oxyrrhis marina* (49), which, admittedly, would have been appropriate to include in our experiments.

It should be noted that three of the bacterial isolates were from the brackish Baltic Sea (*Flavobacterium* sp. BAL38, *Pedobacter* sp. BAL39, and *Thalassobaculum* sp. BAL199; Table 1) with salinity levels more akin to freshwater lakes than to oceans, and these three bacteria showed interactions similar to the fully marine bacteria, including lysis of one or more protists and maintained viable populations in the presence of the protists (in the media adapted to the protists). Importantly, most bacterial isolates were also able to grow axenically in protist-adapted media despite low salinity levels. Virulence factors such as type III–VI secretion systems are widely distributed across bacterial phyla and classes (50), and virulent freshwater *Vibrio* spp. have been found (our study included two *Vibrio* isolates) (51). Together, this suggests that the marine bacteria in our study were compatible with environmental conditions preferred by the model protists. Ultimately, we made the judgment that it was more important to be able to compare our results to the medical microbiology literature than to consider if these precise organisms encounter each other in nature. Thus, not all of our results can be directly extended to the marine environment *in situ*. Future studies are needed that specifically target predatory protists naturally found alongside marine bacteria.

### Bacteria lysed protists in a selective and density-dependent manner

Lysis of protists during bacterial growth was observed with 10 of the studied bacteria. Our survey revealed pronounced differences in how vulnerable the three protists were to bacterially mediated lysis, with *A. polyphaga* being most sensitive, and *T. pyriformis* and *E. gracilis* also differing in their modes of responses. For example, *E. gracilis* showed stress responses by rounding up or by altering its pigmentation. The reasons for the increased pigmentation in response to bacterial exposure in some cases is unclear. It is possible that a shift in pigment composition occurred, such as an increased carotenoids-to-chlorophyll ratio, which has been observed in green algae under stress conditions like nitrogen limitation or high light intensity (52). In contrast, other studies have identified algicidal compounds produced by marine bacteria that instead decrease pigmentation, followed by cell rupture, of various algae species. These reactions are possibly connected to increased levels of reactive oxygen species (10, 11). Because *E. gracilis* has a mixotrophic lifestyle, it may not be as sensitive to toxic extracellular compounds that target the photosynthesis system (10), which could, in part, explain its atypical reaction.

In the cases where lysis of protists was observed, bacterial lysis efficacy was highest in the treatment with the higher bacteria:protist inoculation ratio. This efficacy was displayed both with respect to if lysis occurred or not and with respect to the time it took until lysis (with longer time required for co-cultures with low initial bacteria:protist ratio). The bacterium *Stenotrophomonas* sp. SKA14 (Gammaproteobacteria) showed the broadest lysis ability, being capable of killing all three protists included in the study at both high and low bacteria:protist inoculation ratios (Table 1; Fig. 1). A more limited lysis ability was displayed by, for example, *Pedobacter* sp. BAL39, which only lysed *A. polyphaga*. Still, BAL39 was highly efficient in lysing the amoeba, as seen from its ability to lyse amoebae at both high and low bacteria:protist ratios. Our survey shows that lysis efficacy on particular protists varies considerably between bacteria in a species-specific manner and that bacteria show distinct differences with respect to the range of protists they can lyse. Bacteria growing in high densities may form biofilms that are typically more tolerant to antimicrobial agents (53) and may result in cytotoxic effects on protist grazers (22). Other bacteria found within aquatic environments have also been shown to lyse protists (10, 20, 21, 54). Our results generally agree with the findings in these reports that lysis occurs within 3–4 days of co-cultivation with pathogenic bacteria. Similar to strain-specific grazing of protists (55), predatory bacteria too may display strain-specific lysis of protists (9, 54).

The mechanism of bacterial-mediated lysis of protists differs, where some bacteria are reproducing intracellularly (20, 21, 56, 57) and disrupting intracellular components (58) until the protist bursts. In other taxa, lysis occurs through extracellular toxic compounds secreted by the bacteria (10). The latter seems to be the preferred mode with algicidal bacteria although lysis as a result of direct contact of algicidal bacteria also occurs (13). Potential virulence mechanisms in our study are discussed in more detail below.

### Simultaneous maintenance of bacterial and protist viability

Simultaneous long-term protist viability and bacterial survival was observed with 14 of the 18 studied bacteria with at least 1 of 3 protists; 24 out of 54 interactions in total, of which 4 bacterial strains displayed intracellular presence within *A. polyphaga*. Indeed, aquatic prokaryotes have developed numerous strategies to avoid predation (59). For example, bacteria may take “shelter” within cellular compartments of the amoeba, protecting them from degradation (22). Alternatively, viable bacteria can be detected in co-cultures because they manage to survive at abundances below a threshold at which grazing is efficient. Indeed, despite efficient initial grazing of marine bacteria by *A. castellanii*, bacteria could still be identified 48 h post infection although at low levels (22). These findings on the ability of bacteria to survive for several days to weeks together with protists indicate that mechanisms for escaping protist grazing are widespread among marine bacteria.

A very complex example of coinciding protist and bacterial viability, which could be considered a mix between protist lysis by bacteria and simultaneous protist viability and bacterial survival, was observed in co-cultures of *Flavobacterium* sp. BAL38 (Bacteroidetes) and *A. polyphaga*. This bacterium was capable of lysing both *A. polyphaga* and *E. gracilis* (Table 1), but the growth curve experiments with amoeba co-cultures showed that the amoeba transiently recovered when BAL38 reached the stationary growth phase (Fig. 3). Thus, bacterial growth was initially delayed by amoeba grazing, but eventually, bacterial growth would affect amoeba viability negatively. Finally, once the bacterial stationary phase was reached, the amoebae were given an opportunity to recover. This indicated that BAL38 can escape predation by protists and may be virulent to such species during proliferation even though they may coexist when the bacteria are in stationary phase. This in some respects mirrors the “Jekyll-and-Hyde” relationship between the coccolithophore *Emiliania huxleyi* and its symbiotic bacterial partner *Phaeobacter gallaeciensis. P. gallaeciensis* first promotes the growth of *E. huxleyi*, only to transition into a pathogenic phase once *E. huxleyi* reaches higher densities (60). Similarly, a *Microbulbifer* strain showed increased algicidal effects against various algae species when at stationary phase as compared to exponential phase (10). Collectively, these findings substantiate the diverse and complex relations found between bacteria and phagotrophic and photosynthetic protists.

### Bacteria utilizing protist-derived nutrients under low-nutrient condition

*Stenotrophomonas* sp. SKA58 was the most notable example of a bacterium that could utilize the protists to gain nutrients for proliferation under low-nutrient conditions (Fig. 3). Interestingly, in nutrient-rich medium, SKA58 was also capable of lysing *A. polyphaga* although, at the same time, a fraction of the amoeba cells remained viable and dividing while inhibiting bacterial growth. Under low-nutrient conditions, however, SKA58 could grow in co-culture and assimilate amoeba-derived nutrients, probably by lysing amoeba cells (as indicated by the temporary drop in viable amoeba despite bacteria being grazed to low levels). The survival efficacy of SKA58 was, however, not dependent on viable amoeba cells, indicating that SKA58 is more likely to utilize the amoeba as a food resource rather than for a symbiotic or commensal interaction. It is also possible that the amoeba cells do not prey on SKA58 as efficiently under low-nutrient conditions as when nutrients are easily accessible, facilitating bacterial consumption of amoeba-derived nutrients. These results indicate a fine balance in “the battle of eating or being eaten,” and that the result of this interaction is strongly affected by nutrient status. Similarly, *Francisella* spp. display enhanced survival in low-nutrient media when co-cultured with *A. polyphaga* or *A. castellanii* (24, 25), possibly as a result of amoeba-excreted compounds (25). If displayed in natural aquatic environments, the combination of using a protist species to gain nutrients when no other nutrients are available and the ability to lyse the same protist would indicate a largely unexplored flow of nutrients directly from eukaryotes to bacteria.

### Viable protists required for bacterial growth

We were intrigued to find that 6 of our studied bacteria species were dependent on viable amoebae to grow but were unable to proliferate in nutrient-rich medium in monoculture (e.g., *Photobacterium* sp. SKA34). These bacteria possibly proliferate intracellularly, as they were observed within *A. polyphaga*. Notably, none of these strains caused amoeba lysis at a 1:1,000 bacteria:amoeba ratio, which was used for this experiment, suggesting an endosymbiotic relationship. Four of these bacteria required live amoebas to proliferate which suggest that their growth was more likely stimulated by amoeba-secreted compounds than by consumption of amoeba cell structures or content. Non-lytic, intracellular obligate bacteria within various amoeba species have been documented (2, 6, 57, 61). Bacteria may eventually be packed in vesicles and ejected from their hosts (22, 62), which could explain their presence in the extracellular environment. It is also possible that bacteria can escape grazing and grow extracellularly, supported by compounds secreted by the amoebae that facilitates growth (25). The latter strategy may be akin to “microbial gardening” where protists deliberately enhance bacterial growth to increase their own food supply (63).

### Protists as evolutionary training grounds for bacterial virulence mechanisms

Bacterial capability to escape predation, lyse protists, and benefit from co-culturing with protists could, in many cases, be caused by expression of well-known virulence gene homologues, which have been shown to be widespread in marine bacteria, including many of the bacteria in our study (30). As an example, type VI secretion system (T6SS) genes were found in eight bacterial strains—all capable of causing lysis and/or interacting in some way favorably with amoebae. T6SS is strongly associated with virulence, but T6SS loci are also highly conserved in non-pathogenic bacteria, suggesting other functions than pathogenesis; this could include commensal relationships between bacteria and eukaryotes and social interactions between bacteria (64). In a recent study, T6SSs of pathogenic *Fransciella* spp. did not affect the interaction with various *Acanthamoeba* spp. (25). *Marinomonas blandensis* MED121 of our study is an example of this type of non-pathogenic, non-dependency relationship with protists. Similar to the type VI secretion system, type III–V (T3SS-T5SS) systems are also associated with bacterial virulence and eukaryotic infections (65). While not as prevalent as T6SS, six strains used in this study possess one or more of these systems (30). Except for *Roseobacter* sp. MED193, all of these strains caused lysis. In contrast, three bacterial strains (*Dokdonia donghaensis* MED134, *Flavobacterium* sp. BAL38, and *Pedobacter* sp. BAL39) were capable of lysing protists without carrying any known virulence gene homologs (30). However, it should be emphasized that there may also be other virulence genes present in the genomes of these bacteria, which were not targeted in the Persson et al. (30) study. Nevertheless, we find it likely that widespread virulence gene homologs in marine bacteria, in many cases, may have a primary function in commensal or virulent interactions with bacterivorous protists. The pathogenic effects in humans associated with these genes have likely evolved long prior to human contact, perhaps as a result of interactions with protists, as indicated by the presence of T3SS in the predicted chlamydiae ancestor about 700 million years ago (66). The finding that the primary function of T3SSs in the environmental and opportunistic pathogen *Pseudomonas aeruginosa* is to kill biofilm-associated amoebae supports this hypothesis (67). Among the secretion systems, most research has focused on T3SS, likely because of its often found association with disease outbreaks of aquatic animals (32). Incidentally, both bacteria that harbored T3SS were able to induce lysis in two (*V. campbellii* AND4) or all three (*Sphingomonas* sp. SKA58) protists.

It has been suggested that protists act as “training grounds” for pathogenic bacteria, which then go on to infect secondary species such as humans (68). Alveolar macrophages, in particular, bear many similarities to phagotrophic protists and are susceptible to infection by pathogenic bacteria, which possibly contributes to the symptoms in respiratory diseases (15). This phenomenon has also been described as “coincidental evolution,” and it’s been shown that grazing-resistant *E. coli* are more likely to harbor virulence factors (69). Thus, because neither horizontal nor vertical transmission occurs for many pathogenic bacteria in humans, coincidental evolution may explain their prevalence in secondary hosts. Commonly cited examples of pathogenic bacteria associated with, specifically, *Acanthamoeba* spp., include *Legionella* spp. and *Mycobacterium* spp. (14). These pathogens display enhanced infectivity when affiliated with amoebas (14). However, as outlined earlier, because the bacteria and protists used in our study are not necessarily found in the same environment, and only a few of them are recognized as human pathogens, the generality of our results needs to be interpreted with care. Given that our studied bacteria grow in rich media, they may be considered to some degree as copiotrophs. With the exception of *Polaribacter* sp. MED152 and *Flavobacterium* sp. BAL38 that are widespread and abundant in the surface ocean, the bacteria we studied are relatively rare and, thus, not representative of the broader oceanic microbiota (70). Yet they may be more prevalent under specific conditions, such as phytoplankton blooms. Copiotrophs are often easily cultivated, making them well-suited for experimental work, and they have been subject to much important ecological research (e.g., [71, 72]). Furthermore, because oligotrophs have streamlined genomes, they are not expected to possess extensive arrays of virulence genes that enable them to inflict damage on protists. From this perspective, we think our selection of bacteria from a variety of distinct taxonomic groups is appropriate and offers novel insights into what such bacteria may do during periods when they potentially flourish and bloom. Lastly, beyond the ecological importance, our results may also have pathological and epidemiological implications in the field of medical microbiology, offering a deeper understanding of protist-bacteria interactions that could affect secondary species, not just the primary protist.

### Conclusions

In conclusion, our results indicate that marine bacteria may be more commonly capable of escaping protist grazing and, in several cases, even kill protists, than previously recognized. In addition, some of these bacteria may also benefit from, or even require, interactions with viable protists in order to thrive and survive. To mediate bacteria-favorable interactions between bacteria and protists may be one of the primary functions of widespread virulence genes in marine bacteria. These complex interactions are likely to be influenced by environmental factors (e.g., temperature and nutritional status) and the physiological status and abundance of the bacteria or protists. Given the ubiquity of temporal dynamics in the composition of natural bacterial and protist communities, the diversity of interactions uncovered here has important implications for how the strength and direction of interactions between bacteria and protists evolve. For example, one could envision a scenario where a sudden release of organic material would trigger the growth of a bacterial population with high capacity to lyse the dominant protists at the time, which would potentially alter the overall grazing pressure on the bacterial community. Thus, the ecological consequences, evolutionary effects, as well as potentially pathogenic and epidemiological potentials of these interactions need to be further elucidated.

## MATERIALS AND METHODS

### Bacterial strains

A total of 18 bacterial strains of marine origin from the Linnaeus University culture collection, Kalmar, Sweden, were cultured either in liquid medium or on agar plates, dependent on experiment, constituted of three different media. These bacterial strains can be characterized as either “moderate” or “extreme” copiotrophs (34). Marine broth 2216 (MB) (Difco) was used for culturing of eleven strains (eight strains from the Mediterranean Sea [MED92, MED121, MED134, MED152, MED193, MED217, MED222, MED297], and one strain each from the Andaman Sea [AND4], the Red Sea [RED65], and the Southern California Bight [SCB49]). ZoBell medium with Skagerrak seawater was used for the four strains isolated from the Skagerrak (SKA14, SKA34, SKA53, and SKA58). ZoBell medium with Baltic seawater was used for the three strains isolated from the Baltic Sea (BAL38, BAL39, and BAL199). Marine agar 2216 (Difco) was used for strains grown in Marine broth. SKA and BAL media were prepared with ZoBell broth, 5 g of peptone (Bacto Peptone; BD) and 1 g of yeast extract (Bacto Yeast Extract; Difco) in 800 mL of Skagerrak or 800 mL Baltic seawater, and 200 mL MilliQ water. For agar plates, 15 g of agar (Difco Bacto Agar) was added to 1,000 mL of medium (SKA and BAL). For estimation of growth rates, one colony of each of the 18 bacterial species was harvested from agar plates and added to 50 mL of MB, SKA, or BAL. Samples were incubated at room temperature (RT) and monitored once every day by measuring optical density (OD). The measured growth rates were then used to harvest bacteria in late exponential growth phase.

### Protists

Three species of protists were used in the study: *Acanthamoeba polyphaga* (strain Linc Ap-1), *Euglena gracilis* (strain CCAP 1224/5Z), and *Tetrahymena pyriformis* (strain provided by Prof. Agneta Andersson, Umeå University, Sweden). *A. polyphaga* has been isolated from a variety of environments, including marine water (36), while the latter two are freshwater protists although *T. pyriformis* have been isolated in salt marshes (38, 40). Trophozoites of *A. polyphaga* were maintained aerobically at 27°C in peptone yeast glucose (PYG) medium, at the bottom of 25 cm^2^ culture flasks, as previously described (48). *T. pyriformis* was grown in peptone-yeast medium (PPY) at RT in the dark. *E. gracilis* were grown in *Euglena gracilis* Medium 1:1 with Jaworski’s Medium (EG:JM) in a north-facing window according to the manuals from CCAP (Culture Collection of Algae and Protozoa, Scotland, United Kingdom). For the different experiments, protist species were grown to a density of 10^6^ cells/mL and then inoculated either in 12-well culture plates (all protists) or added to 5 mL glass tubes (*Acanthamoeba* used in Fig. 3) for experiments measuring optical density.

### Bacterial co-culturing with protists

Protista species were grown in respective protist media and inoculated with the 18 different bacteria species grown in respective liquid bacterial media at a high (10:1 bacteria:protista) and low (1:1,000 bacteria:protista) bacterial ratios using 12-well culture plates to observe consequences of co-cultivation on protists (used for Table 1). Additional 12-well plates for each of the three protista were inoculated with a high bacteria:protista ratio (10:1) to estimate the survival of bacteria in protist co-cultures. Cultures of only the different unicellular protista or bacteria alone in protist media maintained in separate plates were used as controls. These bacterial monoculture controls were especially important to confirm that the marine bacteria were able to grow and thrive in non-native (protist media) cultures. Co-cultures and controls were incubated at RT for 15 days. Daily observations of co-cultures with invert microscopy (Leica Microsystems, Germany, ×400 magnification) were used to determine protista cell morphology and viability. Moreover, samples were taken every third day and plated on agar plates for the presence of live and growing bacterial cells, to obtain estimates of bacterial survival expressed as growth or no growth. This, at the same time, allowed repeated evaluation of the identity of bacteria through the experiments by verification of their distinct colony morphologies (e.g. differences in color, texture, and size). All cultures were performed in triplicates. Results were presented as average survival time based on two independent experiments. Observations of intracellular bacterial location in *A. polyphaga* were performed using phase contrast microscopy (Leitz DIALUX 20 EB, Germany, ×1,000 magnification), and differentiation was made between bacterial presence in vacuoles or in cytoplasm.

### Bacterial survival with *A. polyphaga*

Bacteria were subjected to three different treatments in PYG media: co-culturing with live amoebae, heat-killed amoebae, and a heat-killed suspension of an earlier co-culture with the same bacteria species (used for Fig. 2). As control, bacteria were grown in PYG medium alone. Heat-killed *A. polyphaga* pre-culture was harvested at a concentration of 10^6^ cells/mL, and 1 mL per sample was put in 1.5 mL tubes and boiled for 10 min. Heat- killed pre-co-cultures were prepared by adding 100 µL bacteria solution with a concentration of 10^4^ cells/mL to 12-well amoebae culture plates and left to grow for 48 h. From these plates, samples of 1 mL were added to 1.5 mL Eppendorf tubes and heat killed by boiling samples for 10 min. Live bacteria were harvested from agar plates and diluted in PYG medium to a final concentration of 10^4^ cells/mL, and 100 µL was added to 1 mL of amoeba cultures with different pretreatments (10^6^ protist cells) for each sample in 12-well plates. All bacteria used in co-cultures were harvested during late exponential phase, a period when growth conditions are sub-optimal, which may initiate stress responses. Duplicate samples were incubated at RT, and 100 µL fractions was taken after 24 h and then once every second day for plating on agar plates for bacterial survival test (growth or no growth) and confirmation of bacterial identity. Plates were also observed microscopically to see changes in amoebae morphology.

### Bacterial growth during co-culturing with *A. polyphaga*

Bacterial samples were taken in approximately late exponential growth phase and added to glass tubes with 5 mL PYG or phosphate buffered saline (PBS), and with or without *A. polyphaga*, at a low concentration, 10^3^ cells/mL at a ratio of 1:1,000; bacteria:protista (used for Fig. 3). Samples were incubated at RT for 13 days and monitored daily by measuring OD and observing in the microscope. Background (media alone or amoeba monocultures, respectively) was subtracted from experimental values. Bacterial growth and survival were confirmed both by microscopic observation and by plating on agar every second day. Amoebae viability in PYG was determined using the trypan blue exclusion test, initially after one day and thereafter every second day. The cells were suspended by brief vortexing and mixed with 0.04% trypan blue (Sigma-Aldrich, St. Louis, MO). The viability was determined by calculating the ratio of stained vs unstained cells in a Bürcher chamber.

## SUPPLEMENTAL MATERIAL

Supplemental material aem.00929-25-s0001.docx Figures S1 and S2.

ASM does not own the copyrights to Supplemental Material that may be linked to, or accessed through, an article. The authors have granted ASM a non-exclusive, world-wide license to publish the Supplemental Material files. Please contact the corresponding author directly for reuse.
