# Supplementary material for: Experimental assessment of interactions between marine bacteria and model protists: from predator-prey relationships to bacterial-mediated lysis
Source: Appl Environ Microbiol. 2025 May 30;91(6):e00929-25. doi: 10.1128/aem.00929-25 (PMC12175539; doi:10.1128/aem.00929-25)
Supplement: Supplemental material — Figures S1 and S2. [file aem.00929-25-s0001.docx]

Supplemental material

**Experimental assessment of marine microbial interactions: from predatory protists promoting bacterial survival to bacterial lysis of the protists**

Diana Axelsson-Olsson, Nikolaj Gubonin, Stina Israelsson and Jarone Pinhassi

**Supplemental figures**


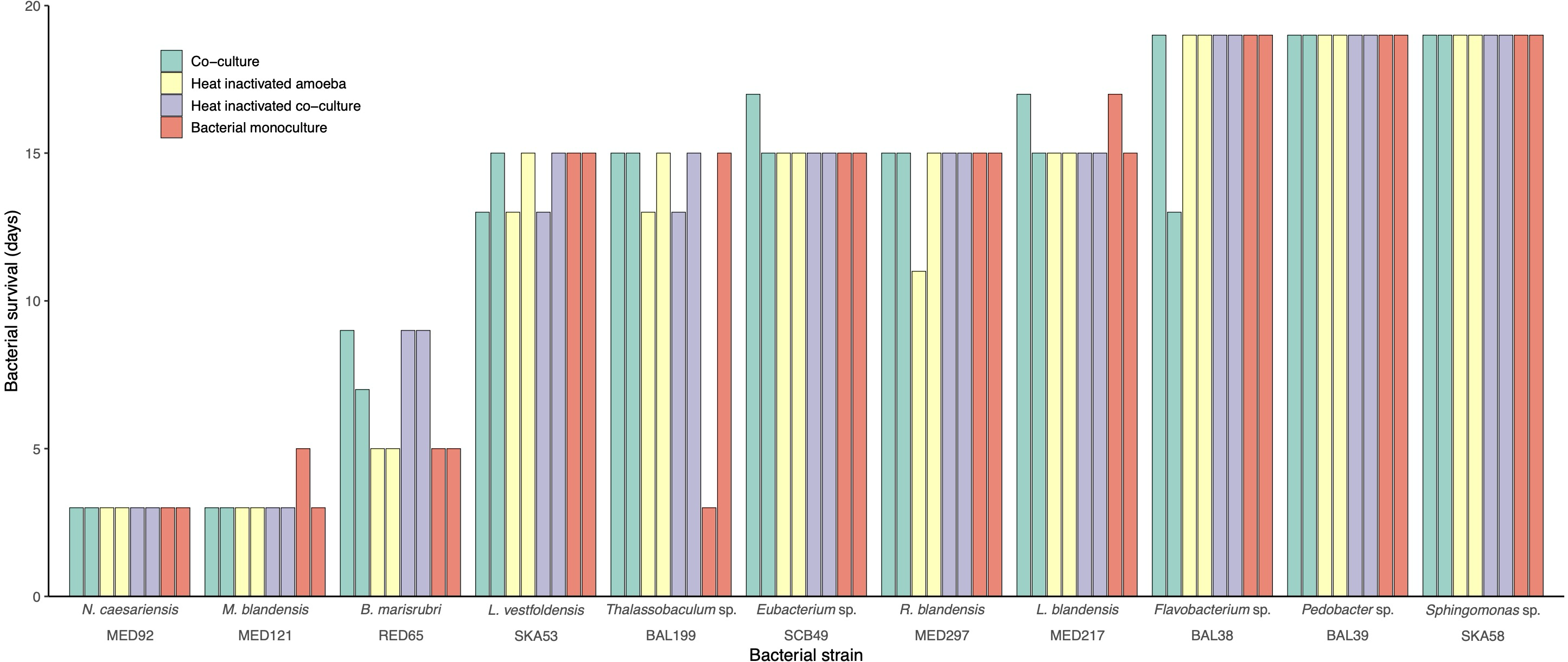


**Fig. S1.** Bacterial survival of the eleven strains not included in Fig. 2, measured for a maximum of 19 days in monoculture and co-culture with amoeba in different settings. Bacteria were cultured in the presence of viable *A. polyphaga* (co-culture), heat-inactivated *A. polyphaga* or a heat-inactivated previous co-culture of *A. polyphaga* and the same bacterial strain and compared to bacteria growing in monoculture.


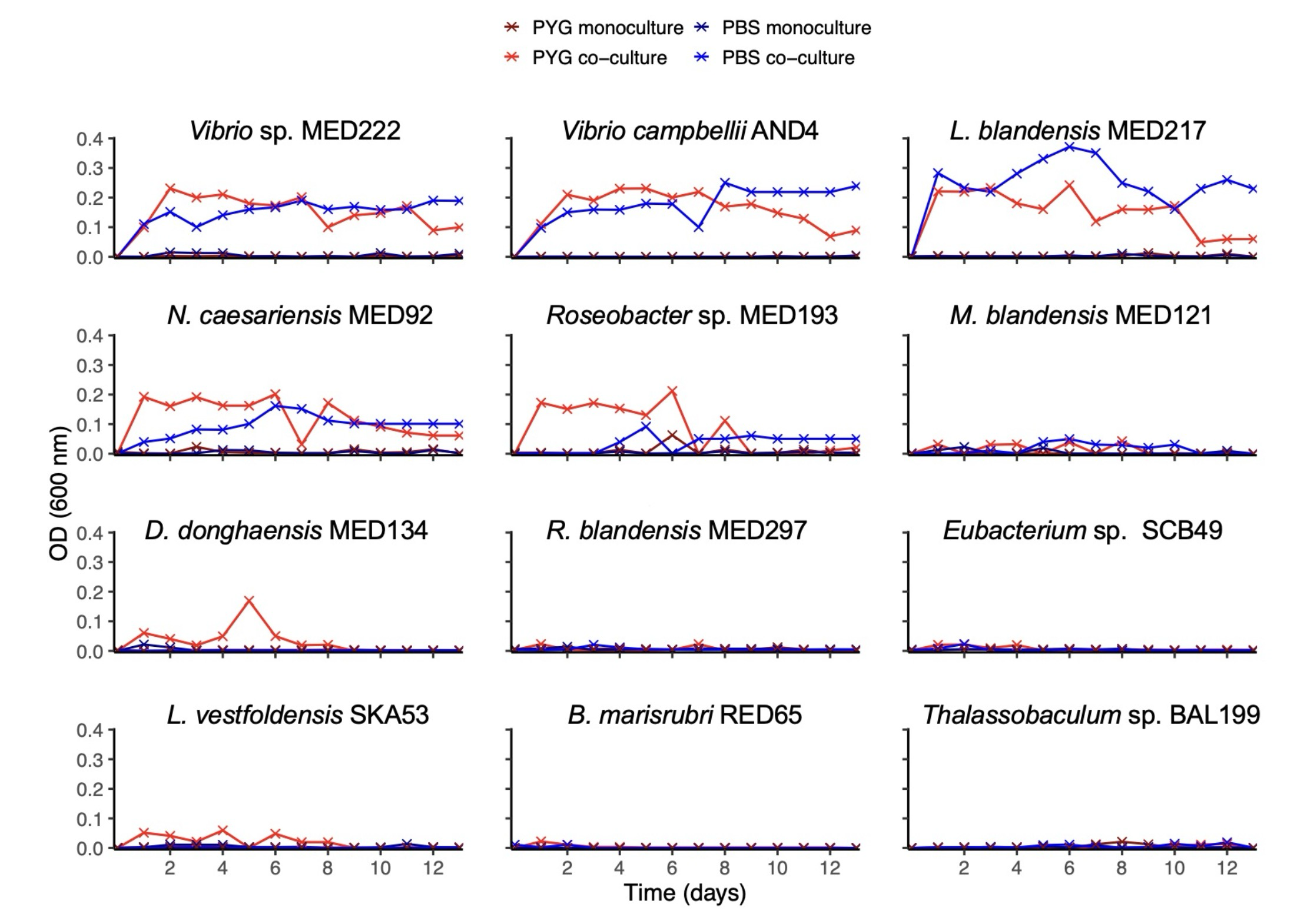


**Fig. S2.** Growth curves of the remaining 12 marine bacterial strains not included in Fig. 3. The bacteria were cultivated in nutrient-rich media (peptone yeast glucose [PYG]), low-nutrient media (phosphate buffered saline [PBS]), and in mono- and co-culture with *A. polyphaga*. All bacteria cultures started from late exponential growth phase and the growth was determined by plating. See Table 1 for full species names.
